# Establishment of a novel clear cell sarcoma cell line (Hewga-CCS), and investigation of the antitumor effects of pazopanib on Hewga-CCS

**DOI:** 10.1186/1471-2407-14-455

**Published:** 2014-06-19

**Authors:** Hidetatsu Outani, Takaaki Tanaka, Toru Wakamatsu, Yoshinori Imura, Kenichiro Hamada, Nobuhito Araki, Kazuyuki Itoh, Hideki Yoshikawa, Norifumi Naka

**Affiliations:** 1 Department of Orthopaedic Surgery, Osaka University Graduate School of Medicine, 2-2 Yamadaoka, Suita, Osaka 565-0871, Japan; 2 Musculoskeletal Oncology Service, Osaka Medical Center for Cancer and Cardiovascular Diseases, 1-3-3 Nakamichi, Higashinari-ku, Osaka 537-8511, Japan; 3 Department of Biology, Osaka Medical Center for Cancer and Cardiovascular Diseases, 1-3-3 Nakamichi, Higashinari-ku, Osaka 537-8511, Japan

**Keywords:** Clear cell sarcoma, Cell line, Xenograft, Pazopanib, HGF, cMET

## Abstract

**Background:**

Clear cell sarcoma (CCS) is a therapeutically unresolved, aggressive, soft tissue sarcoma (STS) that predominantly affects young adults. This sarcoma is defined by t(12;22)(q13;q12) translocation, which leads to the fusion of Ewing sarcoma gene (*EWS*) to activating transcription factor 1 (*ATF1*) gene, producing a chimeric *EWS-ATF1* fusion gene. We established a novel CCS cell line called Hewga-CCS and developed an orthotopic tumor xenograft model to enable comprehensive bench-side investigation for intensive basic and preclinical research in CCS with a paucity of experimental cell lines.

**Methods:**

Hewga-CCS was derived from skin metastatic lesions of a CCS developed in a 34-year-old female. The karyotype and chimeric transcript were analyzed. Xenografts were established and characterized by morphology and immunohistochemical reactivity. Subsequently, the antitumor effects of pazopanib, a recently approved, novel, multitargeted, tyrosine kinase inhibitor (TKI) used for the treatment of advanced soft tissue sarcoma, on Hewga-CCS were assessed *in vitro* and *in vivo*.

**Results:**

Hewga-CCS harbored the type 2 *EWS-ATF1* transcript. Xenografts morphologically mimicked the primary tumor and expressed S-100 protein and antigens associated with melanin synthesis (Melan-A, HMB45). Pazopanib suppressed the growth of Hewga-CCS both *in vivo* and *in vitro*. A phospho-receptor tyrosine kinase array revealed phosphorylation of c-MET, but not of VEGFR, in Hewga-CCS. Subsequent experiments showed that pazopanib exerted antitumor effects through the inhibition of HGF/c-MET signaling.

**Conclusions:**

CCS is a rare, devastating disease, and our established CCS cell line and xenograft model may be a useful tool for further in-depth investigation and understanding of the drug-sensitivity mechanism.

## Background

Clear cell sarcoma (CCS) of tendons and aponeuroses is a rare, malignant, soft tissue tumor [1] characterized by melanocytic differentiation, including immunohistochemical positivity for melanocyte specific-microphthalmia-associated transcription factor (M-MITF), S100 calcium binding protein (S-100), Melan-A, and melanoma-associated antigen human melanoma black 45 (HMB45) [2]. Typically, CCS arises in the extremities of young adults and accounts for approximately 1% of all soft tissue sarcomas (STSs) [2]. It usually appears as a deep-seated, slowly growing mass, and approximately 50% patients develop lung or nodal metastases [2]. Because CCS is very resistant to conventional chemotherapy and radiation therapy, the 5-year overall survival is reported to be only 30%–67% [3-11]. Cytogenetic analysis of CCS has detected the presence of clonal chromosomal translocation, t(12;22)(q13;q12), and identified the fusion of the *ATF1* and *EWS*, resulting in the *EWS-ATF1* fusion gene [12,13]. Several types of fusion transcripts have been described, of which the most common result from the fusion of exon 8 of *EWS* with exon 4 of *ATF1* (type 1), followed by the fusion of exon 7 of *EWS* with exon 5 of *ATF1* (type 2) and the fusion of exon 10 of *EWS* with exon 5 of *ATF1* (type 3) [14]. The rarity of the disease makes it difficult to conduct a clinical study to test the efficacy of a novel therapy. Therefore, we thought it was important to develop a CCS experimental model for understanding the molecular determinants of CCS and developing therapeutic strategies.

Pazopanib is a novel, orally available, multitargeted, TKI targeting several tumor and tumor environment factors with high affinity against vascular endothelial growth factor receptor (VEGFR)1, VEGFR2, and VEGFR3 and low affinity against platelet-derived growth factor receptor (PDGFR)α, PDGFRβ, fibroblast growth factor receptor (FGFR)1, FGFR2, and stem cell factor receptor (c-Kit) [15]. A phase III trial conducted to assess the efficacy and safety of pazopanib for metastatic STS using placebo as a control demonstrated a statistically significant improvement in progression-free survival [16], leading to approval of this drug for the treatment of advanced STSs as the first molecular targeted agent in Japan. However, in the phase III study, no detailed information about CCS was available, and there have been no reports demonstrating the treatment effects of pazopanib against CCS. To date, a small number of CCS cell lines have been successfully established [17-27], but those harboring disease specific *EWS-ATF1* fusion gene and available in both *in vitro* and *in vivo* study are quite rare. Thus, we established a new CCS cell line, Hewga-CCS, and investigated the antitumor effects of pazopanib on Hewga-CCS *in vitro* and *in vivo*.

## Methods

### Establishment of Hewga-CCS

The clinical course of the patient with CCS was described in the Supplementary Information (Additional file 1: Figure S1). Tumor cells were isolated from surgically resected tissues obtained from excised skin metastatic lesions after the patients provided written informed consent. The study of establishment was conducted in accordance with the guidelines of the Ethics Committee of Osaka Medical Center for Cancer and Cardiovascular Diseases. The tumor tissues were minced and incubated with 1 mg/mL of collagenase (Sigma–Aldrich, St. Louis, MO, USA) for 1 h at 37°C. Cell suspensions were passed through a 40-μm nylon mesh (BD Falcon, Franklin Lakes, NJ, USA), and the tumor cells were cultured in Dulbecco’s modified Eagle’s medium (DMEM; Life Technologies, Carlsbad, CA, USA) with 10% fetal bovine serum (FBS; MP Biomedicals, Aurora, OH, USA). The adherent cells were maintained for >36 months in culture and passed >200 times, which fulfilled the criteria of a cell line. Throughout the establishment of this cell line, the attached cells continuously expressed the *EWS-ATF1* transcript (data not shown).

### Chromosomal analysis

Metaphase chromosome spreads from Hewga-CCS cells were prepared according to standard procedures. Hewga-CCS cells were treated with 20 μg/ml of colcemide overnight and harvested. After treatment of 0.075 M KCl for 20 min at 37°C, cells were fixed 3 times with methanol and acetic acid (3:1) and fixed cells were spread on slides. Multicolor fluorescence *in situ* hybridization (M-FISH) was performed using commercially available M-FISH kits (MetaSystems, Altlussheim, Baden-Württemberg, Germany) according to the manufacturer’s protocol. Briefly metaphase spreads were hardened 70°C for 2 h. After applying M-FISH probes on the metaphase spreads, co-denaturation of target DNA with probe DNA was performed at 70°C for 5 min, followed by 72 h incubation at 37°C to allow hybridization of the probes. The slides were then washed twice with 50% formamide/2 × standard saline citrate (SSC) solution for 20 min at 37°C, 2 × SSC for 10 min at room temperature and 1 × SSC for 10 min. The slides were then counterstained with 4′,6-diamidino-2-phenylindole (DAPI) and mounted. Separate fluorochrome images were captured using a Leica DC 350FX cooled CCD camera (Leica Microsystems, Wetzlar, Hesse, Germany) mounted on a Leica DM600 B microscope using Leica DM600 B software. The images were analyzed using Leica CytoVision (Leica). The chromosomal analyses were examined at passage 110 and 111.

### Enzyme-linked immunosorbent assay (ELISA)

A total of 1 × 10^5^ cells/well were seeded in 6-well plates in triplicate and cultured for 72 h. Quantikine ELISA kits (R&D Systems, Minneapolis, MN, USA) were used in accordance with the manufacturer’s instructions to measure secreted hepatocyte growth factor (HGF) and VEGF levels in supernatants derived from Hewga-CCS or SYO-1, which is a human synovial sarcoma cell line that was kindly provided by Dr. Ozaki (Okayama University, Okayama, Japan).

### Genetic analysis

TRIzol reagent (Life Technologies) was used to purify total RNA. Total RNA (1 μg) was used for the reverse transcription reaction with the High Capacity cDNA Reverse Transcription kit (Life Technologies) according to the manufacturer’s instructions. *EWS-ATF1* cDNA was identified by polymerase chain reaction (PCR) using *EWS* forward primer 5′-TCC TAC AGC CAA GCT CCA AGT C and *ATF1* reverse primer 5′-ACT CGG TTT TCC AGG CAT TTC AC. For sequence analysis, the reverse-transcriptase (RT) PCR-amplified *EWS/ATF1* cDNA fragments were analyzed on 1.5% agarose gels, purified using a Qiagen gel extraction kit (Qiagen, Hilden, Germany), and directly sequenced using the dideoxy procedure and an ABI Prism BigDye terminator cycle sequencing ready reaction kit (Life Technologies) with forward or reverse primers (forward ACTGCAACCTATGGGCAGAC; reverse, CTGATTGCTGGGCACAAGTA) on an Applied Biosystems Model 373A DNA sequencing system. BLAST software (http://blast.ncbi.nlm.nih.gov/Blast.cgi) was used for computer analysis of sequence data.

### Cell proliferation assay

Hewga-CCS cells were cultured in DMEM with 10% FBS. A total of 1 × 10^5^ cells/well were seeded in 6-well plates in triplicate. Cell proliferation was measured by cell counts or by using the CellTiter-Glo Luminescent Cell Viability Assay® (Promega, Madison, WI, USA) according to the manufacturer’s protocols. Trypan blue exclusion-based methods were used to determine cell counts. These analyses were examined at passage 120 to 130.

### Phosphoreceptor tyrosine kinase (RTK) array

To evaluate the expression of phosphorylated RTKs, a Proteome Profiler Array Kit (R&D Systems) comprising spotted antibodies for 49 kinase phosphorylation sites was used to perform the phospho-RTK array according to the manufacturer’s protocol.

### Cell cycle analysis

Resuspended Hewga-CCS cells (5 × 10^5^) were plated in DMEM with 10% FBS and grown overnight before treatment with 10 μmol/L of pazopanib or vehicle. After 24 h of treatment, the cells were collected, washed, and stained with propidium iodide (PI) solution (25 μg/mL of PI, 0.03% NP-40, 0.02 mg/mL RNase A, 0.1% sodium citrate) for 30 min at room temperature. A BD FACSCanto II flow cytometer (BD Biosciences, San Jose, CA, USA) was used to analyze the cell cycle.

### Western blot analysis

Cells were scraped and lysed in ice-cold RIPA buffer (Thermo Scientific, Waltham, MA, USA) supplemented with protease/phosphatase inhibitor cocktail (Cell Signaling Technology, Danvers, MA, USA). After centrifugation, the supernatants were collected and a BCA Assay Reagent (Thermo Scientific) was used to determine protein concentrations. Fifty-microgram aliquots of protein were separated by 10% sodium dodecyl sulfate polyacrylamide gel electrophoresis and transferred onto a PVDF membrane. After blocking with 5% skim milk in Tris-buffered saline with 0.1% Tween-20 for an hour, bound proteins were exposed to the following antibodies overnight at 4°C: MET (#8198 rabbit monoclonal; Cell Signaling Technology), p-MET (#3077 rabbit monoclonal; Cell Signaling Technology), β-actin (sc-47778 mouse monoclonal; Santa Cruz Biotechnology, Santa Cruz, CA, USA), Akt (#4691 rabbit monoclonal; Cell Signaling Technology), p-Akt (#4060 rabbit monoclonal; Cell Signaling Technology), Erk (#4695 rabbit monoclonal; Cell Signaling Technology), and p-Erk (#4370 rabbit monoclonal; Cell Signaling Technology). The secondary antibodies used were HRP-conjugated goat anti-rabbit and anti-mouse IgG (GE Healthcare, Little Chalfont, Buckinghamshire, UK). An ECL plus Western Blotting Detection System kit (GE Healthcare) was used to detect western signals.

### RNA interference

Lipofectamine 2000 reagent (Life Technologies) was used according to the manufacturer’s instructions to transfect cells with 20-nM small interfering RNAs (siRNAs). Two kinds of siRNAs against MET were purchased from Cell Signaling Technology (#6618S).

### *In vivo* models

Hewga-CCS cells (1 × 10^7^) were subcutaneously injected into the flanks of 5-week-old athymic nude mice (BALB/c nu/nu; SLC, Shizuoka, Japan). Calipers were used to measure tumor size, and tumor volume was calculated according to the formula (a × b^2^)/2, where “a” was the longest diameter and “b” was the shortest diameter of the tumor. When the tumors reached a volume of palpable size, the mice were randomized and divided into drug-treated and vehicle-treated groups. Pazopanib was kindly provided by GlaxoSmithKline (London, UK), and pazopanib solution was prepared as described previously [15]. Bevacizumab was purchased from Chugai Pharmaceutical Co. Ltd. (Tokyo, Japan). Bevacizumab dissolved in PBS was intraperitoneally injected at 10 mg/kg concentration (200 μg/mouse) twice a week for the indicated times. All experiments were approved by our institutional animal committee (the Institutional Animal Care and Use Committee of Osaka University Graduate School of Medicine) and institutional biosafety committee (Osaka University Living Modified Organism Experiments Safety Committee).

### Histological analysis

Tumor tissue samples were fixed in 10% buffered formalin for 24 h and embedded in paraffin. Hematoxylin and eosin were used to stain 4-μm sections, and serial sections were used for immunohistochemical analysis. The primary antibodies used were anti-Ki67 (M7240; Dako, Glostrup, Denmark), anti-S100 (IR50461; Dako), anti-HMB45 (N1545; Dako), and anti-Melan-A (IR633; Dako). The Liquid DAB + Substrate Chromogen System (Dako) was used according to the manufacturer’s protocol to perform peroxidase staining. An *in situ* apoptosis detection kit (Takara Bio, Otsu, Japan) was used according to the manufacturer’s protocol to perform terminal deoxyribonucleotidyl transferase (TDT)-mediated dUTP-digoxigenin nick end labeling (TUNEL) staining.

### Statistical analysis

The data are shown as averages and standard deviations. Two-tailed Student’s *t*-tests were used to compare the data. The immunohistochemical results were statistically analyzed using Fisher’s exact test. P-values of <0.05 were considered statistically significant.

## Results

### Characterization of the Hewga-CCS cell line

**Figure 1:**
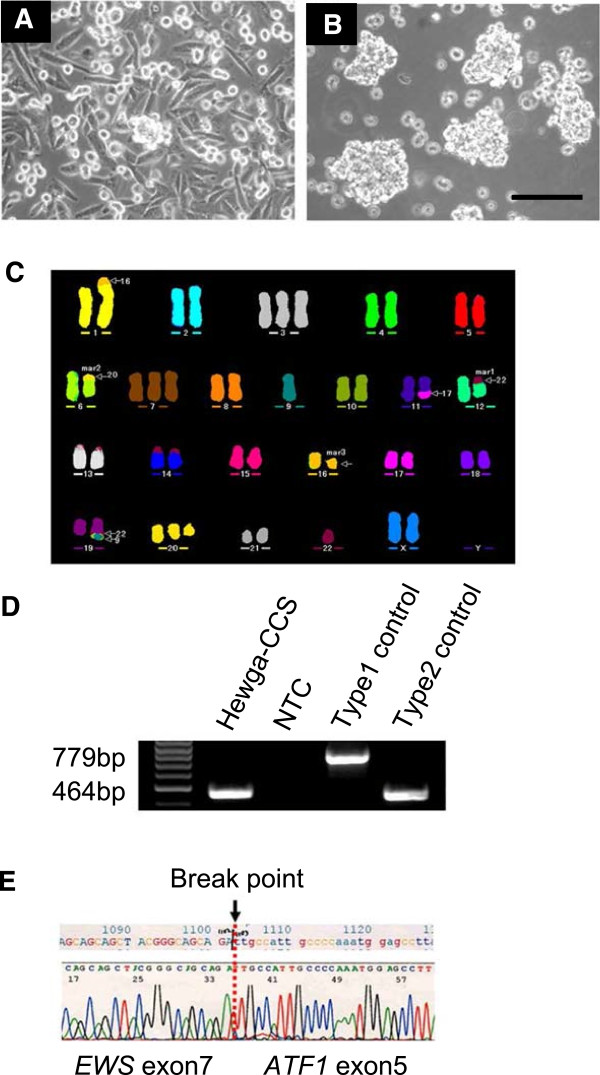
**Morphology and genetic analysis of Hewga-CCS cells. (A)** Phase-contrast image of Hewga-CCS cells cultured in serum-containing medium (DMEM with 10% FBS). Bar: 100 μm. **(B)** Phase-contrast image of Hewga-CCS cells cultured in the low-attachment plates (DMEM with 20% FBS). Bar: 100 μm. **(C)** Representative karyotype of Hewga-CCS. M-FISH analysis showed 5 recurrent structural chromosomal rearrangements: der(1)t(1;16), der(6)t(6;20), der(11)t(11;17), der(12)t(12;22), and der(19)t(9;19;22). **(D)** RT-PCR with *EWS* forward primer located in exon 7 and *ATF1* reverse primer in exon 7 that amplifies a 779 base pair (bp) PCR product in the type 1 *EWS-ATF1* transcript and a 464 bp PCR product in the type 2 *EWS-ATF1* transcript. Hewga-CCS cells and the type 2 EWS-ATF1 transcript control exhibit a single band of approximately 460 bp in lane 1 and lane 4. No band is present for the negative control (NTC) of distilled water in lane 2. The type 1 EWS/ATF1 transcript control exhibits a single band of approximately 780 bp in lane 3. **(E)** A partial sequence chromatogram shows that ATF1 exon 5 was fused with EWS exon 7. The arrow indicates the junction of the ATF1 and EWS genes.

Tumor cells obtained from skin metastatic lesions grew in the form of an adherent monolayer in DMEM with 10% FBS. Two types of cells were obtained: small round cells and polygonal spindle cells (Figure 1A). The doubling time of the cultured cells was approximately 44 h (Additional file 2: Figure S2). To examine the capacity of spheroid formation, we cultured the cells on low-attachment dishes with 20% FBS according to the protocol of our previous study [28]. Under the low-attachment condition, the Hewga-CCS cells began to aggregate and form loose clumps and continued to increase in size; however, they did not form well-rounded structures (Figure 1B).

In chromosomal analysis, a total of 50 metaphase cells from Hewga-CCS were examined by G-banding methods. The following karyotypes were found: 44–47, XX, add(1)(p?36.1), +3, -5, -6, +7, -9, add(11)(q13), -12, -16, +19, add(19)(q?13.1), -20, -22, +mar1, +mar2,+mar3 (Additional file 3: Figure S3 and Additional file 4: Table S1). M-FISH analysis revealed 5 recurrent structural chromosomal rearrangements, including t(12;22) (Figure 1C and Additional file 5: Table S2).

To verify the presence and investigate the type of *EWS-ATF1* chimeric transcripts in Hewga-CCS cells, we performed RT-PCR and direct sequence analyses. RT-PCR with *EWS* forward primer and *ATF1* reverse primers amplified cDNA fragments of the *EWS-ATF1* transcript (Figure 1D). Sequencing of the amplified fragments showed that *EWS* exon 7 was fused with *ATF1* exon 5, which was proven to be the type 2 transcript of *EWS-ATF1* (Figure 1E) [14].

**Figure 2:**
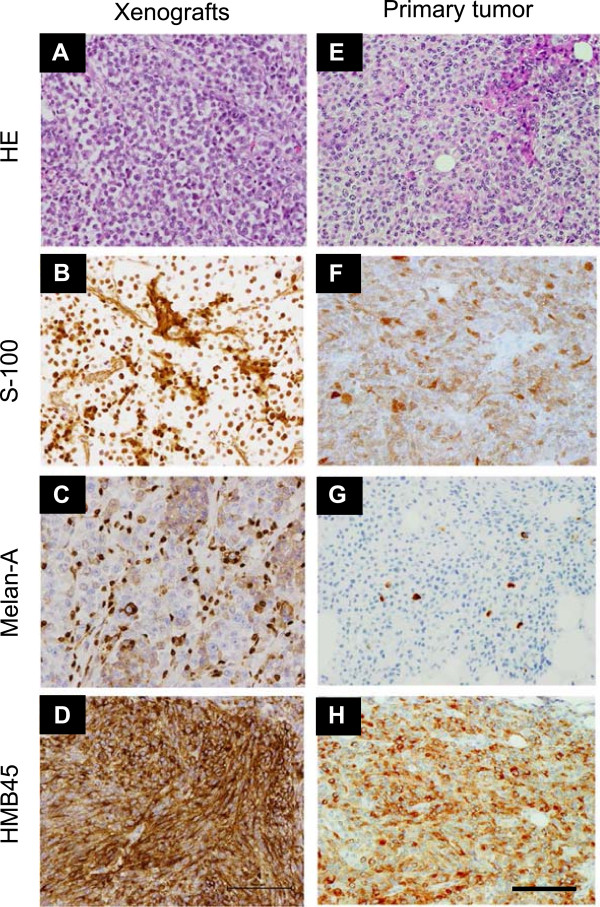
**Histological appearance of Hewga-CCS xenografts and primary tumor. (A, E)** Hematoxylin/eosin (HE) staining and immunohistochemical staining showing expression of **(B, F)** S-100 protein, **(C, G)** Melan-A, and **(D, H)** HMB45 in the Hewga-CCS xenografts and primary tumor. Bar: 100 μm.

To determine tumorigenicity, 1 × 10^7^ Hewga-CCS cells were subcutaneously injected into the dorsal flank of nude mice. All animals developed solid tumors at the sites of injection (Additional file 6: Figure S4). Histological analyses showed that xenografts comprised nests or short fascicles of only slightly polymorphous clear cells, and the nuclei were large and round with low mitotic activity. The morphological features were very similar to those observed in the primary tumor. The positive immunoreactivities of S-100 protein, Melan-A, and HMB45 in the Hewga-CCS xenografts were also similar to those of the primary tumor (Figure 2). These results demonstrated that Hewga-CCS harboring the type 2 *EWS-ATF1* transcript continuously grew *in vitro* and developed tumors in nude mice, while retaining a phenotype similar to that of the primary tumor in terms of cell morphology and melanocytic features.

### Pazopanib inhibited Hewga-CCS cell growth *in vitro*

**Figure 3:**
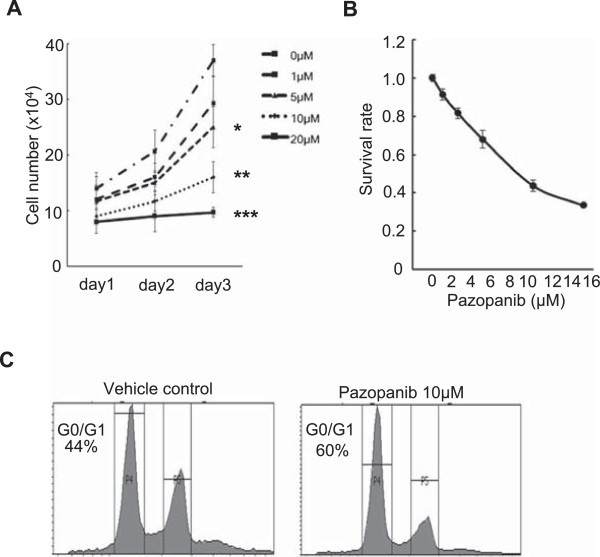
**Antitumor effects of pazopanib on Hewga-CCS cells *in vitro. *(A)** Hewga-CCS cells were cultured in serum-containing medium in the absence or presence of 1, 5, 10, and 20 μM of pazopanib. The cell numbers were counted. Bars: SD. *P < 0.05, **P < 0.01, ***P < 0.001. **(B)** Hewga-CCS cells were treated with pazopanib (0, 1, 2.5, 5, 10, 15 μM) for 72 h, and the survival rates were assessed by CellTiter-Glo®. Bars: SD **(C)** 10 μM of pazopanib- or vehicle-treated Hewga-CCS cells were stained with propidium iodide and analyzed by flow cytometry.

Recent clinical evidence showing that pazopanib was effective for metastatic STS has been published [16]. However, the sensitivity of CCS to pazopanib remains unknown. To test the effects of pazopanib on the growth of Hewga-CCS cells, these cells were incubated for 24 to 72 h with pazopanib at concentrations of 0–20 μmol/L, following which the number of living cells was counted. A dose-dependent decrease in the number of living Hewga-CCS cells was observed (Figure 3A). The IC_50_ value of pazopanib was approximately 8 μmol/L after 72 h of culture (Figure 3B).

Vehicle or 10 μmol/L of pazopanib was used to perform cell cycle analysis. At 24 h of culture, an enhanced G0/G1 peak was observed in the pazopanib-treated cells (Figure 3C). No cleaved caspase-3 protein or cleaved poly-ADP-ribose was detected after culture with pazopanib (data not shown). These data indicated that pazopanib has a direct antiproliferative effect on Hewga-CCS cells *in vitro*.

### The c-MET pathway is a potential target for pazopanib in Hewga-CCS cells

**Figure 4:**
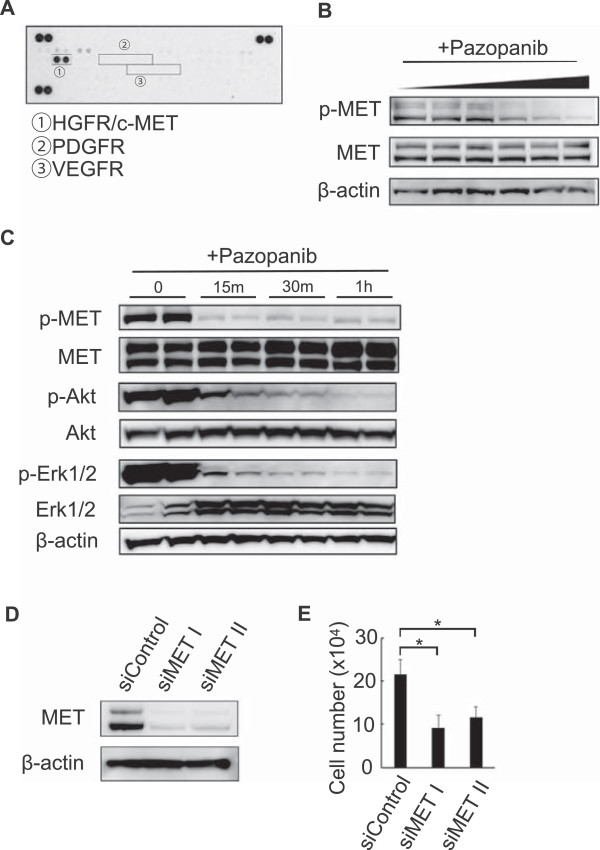
**The c-MET pathway as a potential target of pazopanib in Hewga-CCS cells. (A)** Phospo-RTK array analysis of Hewga-CCS cells. **(B)** Western blot analyses of Hewga-CCS cells treated with increasing doses of pazopanib (0, 1, 5, 10, 15, 20 μM) for 30 min. **(C)** Western blot analyses of Hewga-CCS cells treated with 30 μM of pazopanib. **(D)** Western blot analyses of Hewga-CCS cells 72 h after the inhibition of c-MET by siRNAs. **(E)** Cell counts of Hewga-CCS cells 72 h after the inhibition of c-MET by siRNAs Bars: SD. *P < 0.01.

To decipher the signaling pathway relevant to the antitumor effect of pazopanib on Hewga-CCS cells, we used phospho-RTK array analysis and observed strong activation of the HGF receptor, but not of VEGFR or PDGFR (Figure 4A). Immunoblotting analyses consistently showed c-MET phosphorylation. Interestingly, pazopanib inhibited autophosphorylation of c-MET in a dose-dependent manner, whereas total c-MET remained constant (Figure 4B). We then examined whether pazopanib-mediated c-MET inhibition affected intracellular signaling in Hewga-CCS cells. Decreases in Akt and Erk1/2 phosphorylation occurred concurrently with the decrease in c-MET phosphorylation (Figure 4C). In addition, silencing of c-MET expression by siRNA significantly suppressed the growth of Hewga-CCS cells (Figure 4D, E). To examine whether pazopanib has the similar effects on other sarcoma cell line, we used Asra-Eps [29], which was our established epithelioid sarcoma cell line driven by HGF/c-MET signaling. We found that pazopanib inhibited Asra-Eps cell growth *in vitro* and autophosphorylation of c-MET in a dose-dependent manner (data not shown). These data suggested that pazopanib exerted antitumor effects on Hewga-CCS by abrogating c-MET signaling.

### Bevacizumab had no effect on Hewga-CCS cell growth

**Figure 5:**
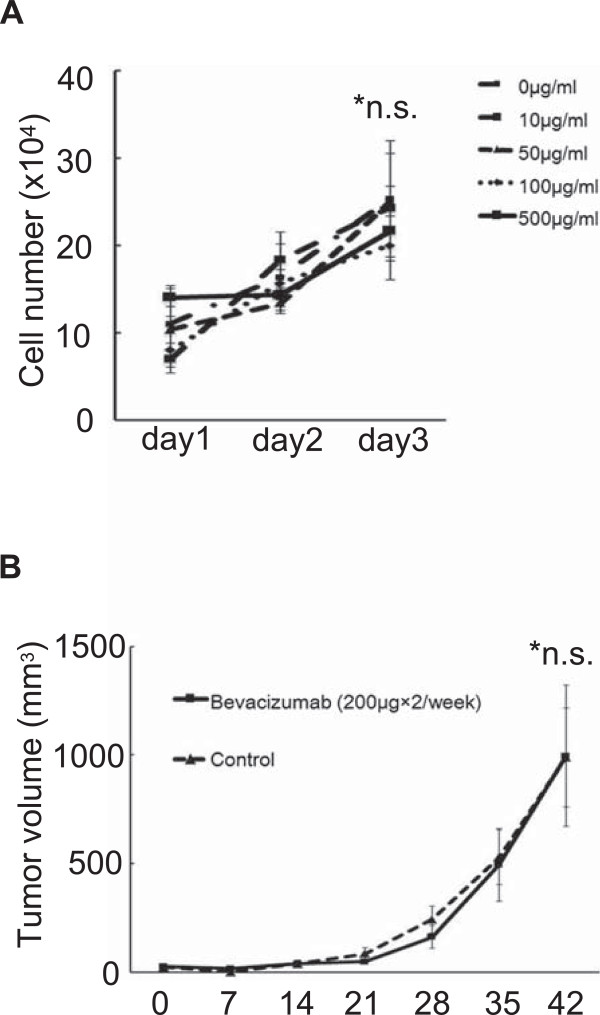
**Bevacizumab had no significant effects on Hewga-CCS cell growth. (A)** Hewga-CCS cells were cultured in serum-containing medium in the absence or presence of 10, 50, 100, and 500 μg/mL of bevacizumab. The cell numbers were counted. Bars: SD. n.s.; not significant. **(B)** Nude mice were inoculated subcutaneously in the flank with 1 × 10^7^ Hewga-CCS cells. When the tumors reached a palpable size, the mice were treated twice per week with intraperitoneal injection of 200 μg of bevacizumab or PBS, following which the tumor size was measured. Bars: SE. n.s.; not significant.

Next, to investigate the potential role of VEGF signaling in Hewga-CCS, we examined the growth of Hewga-CCS cells treated with bevacizumab, humanized murine monoclonal VEGF antibody [30]. Bevacizumab did not affect the proliferation and survival of Hewga-CCS *in vitro* (Figure 5A). We also tested the antitumor effects of bevacizumab on the progression of Hewga-CCS xenografts. Treatment with bevacizumab showed no significant impact on tumor growth (Figure 5B). In addition to the phosphor-RTK array findings indicating no VEGFR activation, these results suggested that VEGF signaling was not crucial for Hewga-CCS cell growth and supported our hypothesis that c-MET signaling was a potential target for pazopanib in Hewga-CCS cells.

### Pazopanib decreased the tumor growth of Hewga-CCS in a xenograft mouse model by suppressing cell cycle progression

**Figure 6:**
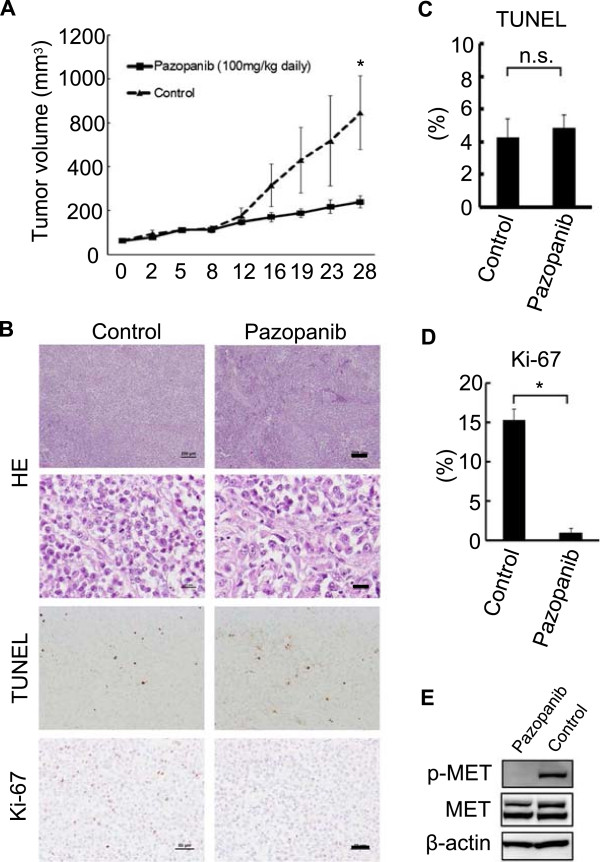
**Pazopanib suppressed tumor growth in a Hewga-CCS tumor xenograft model. (A)** Nude mice were inoculated subcutaneously in the flank with 1 × 10^7^ Hewga-CCS cells. When the tumors reached a palpable size, the mice were treated daily by oral gavage pazopanib (100 mg/kg) or vehicle control, following which tumor size was measured. Bars: SE. * P < 0.05. **(B)** Representative microscopic images of tumor sections are shown to be stained with hematoxylin/eosin (HE), TUNEL, and Ki-67. Bars in the top rows: 200 μm. Bars in the second rows: 20 μm. Bars in the bottom rows: 50 μm. **(C)** The TUNEL assay showed no significant difference between pazopanib- and vehicle control-treated xenografts. n.s.; not significant. **(D)** Ki-67 staining showed significantly suppressed cell cycles in pazopanib-treated xenografts. * P < 0.05. **(E)** Western blot analyses of Hewga-CCS xenografts. Xenografted mice were treated with 100 mg/kg of pazopanib or vehicle control orally once a day for 1 week, sacrificed 3 h after final administration, and subjected to Western blot analyses.

Lastly, we assessed the *in vivo* efficacy of pazopanib using a Hewga-CCS xenograft mouse model. Tumor growth in treated mice was significantly delayed compared with that in the vehicle group (Figure 6A). Consistent with the *in vitro* data, TUNEL assays on tumor sections from treated and control mice showed no significant differences, but Ki-67 staining was significantly decreased in the pazopanib-treated group (Figures 6B–D). We also investigated c-MET activation in Hewga-CCS tumor tissues and found that pazopanib inhibited c-MET phosphorylation in Hewga-CCS xenografts (Figure 6E, Additional file 7: Figure S5). These results demonstrated that pazopanib delayed Hewga-CCS tumor growth by suppressing cell cycle progression, not by inducing tumor cell apoptosis, at least in part through the inhibition of c-MET signaling.

## Discussion

Because of the histological similarities with malignant melanoma, CCS is also known as malignant melanoma of soft parts [31]. However, the genetic findings of the *EWS-ATF1* fusion gene support the supposition that CCS and malignant melanoma are 2 distinct entities [13]. To the best of our knowledge, 12 CCS cell lines have been reported in the English literature [17-27], and there are only 4 cell lines that have been shown to possess both tumorigenicity in immunodeficient mice and *EWS-ATF1* fusion transcripts (Table 1). Furthermore, there is only 1 cell line (UM-CCS-1) that has the type 2 *EWS-ATF1* transcript (Table 1). However, UM-CCS-1 could be passaged only in nude mice. Therefore, Hewga-CCS is the first cell line that harbors the type 2 chimeric *EWS-ATF1* transcript and can be stably cultured *in vitro* and xenografted in nude mice.

**Table 1:** Characterization of clear cell sarcoma cell lines

**Cell lines**	**Age/Sex**	**Site**	**Type of EWS-ATF1**	**Tumorigenicity**
SU-CCS1 (1984)	16/F	heel	1	yes
HS-MM (1993)	39/M	knee	-	yes
NCS-1 (1994)	38/M	foot	-	yes
DTC1 (1995)	-	chest wall	1	-
MST1 (1996)	14/F	knee	-	yes
Kao (1997)	9/F	thigh	1	yes
MP-CCS-SY (2002)	17/F	ankle	1	-
GG62 (2002)	25/F	lower leg	1	-
MST2 (2004)	60/M	knee	1	-
MST3 (2004)	34/M	groin	1	-
CCS292 (2006)	-	-	1	yes
UM-CCS-1 (2002)	60/F	thigh	2	yes (*in vivo* only)
Hewga-CCS	34/F	toe	2	yes

-: Data not described.

It has been reported that *EWS-ATF1* directly activates the melanocyte transcription factor (*MITF)*[26], which in turn activates the c-MET gene [32]. Furthermore, c-MET is widely activated in CCS in an autocrine fashion by its ligand HGF, and CCS strongly depends on HGF/c-MET signaling [33,34]. In agreement with previous reports, we identified a robust activation of c-MET in Hewga-CCS cells (Figure 4A). In addition, we found that Hewga-CCS cells secreted higher amounts of HGF and moderate amounts of VEGF into the culture media compared with the amount of SYO-1 (Additional file 8: Figure S6). These results indicated that Hewga-CCS produced autocrine ligand HGF to activate CCS driver kinase c-MET. Therefore, from the context of analyzing drug sensitivity, Hewga-CCS driven by c-MET signaling commonly observed in CCS can be useful for the accelerated development of targeted therapies for CCS.

Pazopanib is approved for the treatment of advanced renal cell carcinoma and advanced STS by the U.S. Food and Drug Administration [16,35]. However, there have only been a few reports that have demonstrated the molecular mechanism by which pazopanib inhibits the growth of a variety of tumors [15,36-40]. Kumar used a cell-free assay system to show kinase activity of pazopanib and found that pazopanib had an IC_50_ value of 6 μmol/L for inhibiting c-MET activity [15]. This value was much higher than the IC_50_ values of <0.1 μmol/L for pazopanib target kinases, including the VEGFRs, PDGFRs, FGFRs, and c-Kit [15]. Podar demonstrated that pazopanib inhibited multiple myeloma cell growth *in vitro* by inhibiting VEGF signaling at IC_50_ values of 10–30 μmol/L [37]. Paesler demonstrated that pazopanib abrogated the survival of chronic lymphocytic leukemia cells at an IC_50_ of 32.7 μmol/L through VEGF pathway suppression [38]. These studies revealed significant differences in IC_50_ values for pazopanib between cell growth assays and cell-free assays. A potential explanation for this discrepancy is the possibility that kinase activity may be different between living cell and cell-free conditions. In this study, the IC_50_ value of pazopanib in terms of Hewga-CCS cell growth was approximately 8 μmol/L, and comparable concentrations were reportedly achieved after once-daily administration of ≥200 mg pazopanib [41]. It was reported that the combination of pazopanib and lapatinib led to complete inhibition of c-MET by an unknown mechanism, although each of the inhibitors alone had marginal or partial effects [36]. Further, Gotink suggested that low binding affinity of a tyrosine kinase inhibitor to a certain kinase may have a crucial impact on cell signaling, while the same inhibitor with a high binding affinity to another kinase may have no significant effect [42]. We demonstrated the inhibition of c-MET in xenografts treated with pazopanib (Figure 6E). In addition, we showed no significant antitumor effects of bevacizumab *in vitro* and *in vivo* (Figure 5). These results indicated that pazopanib delayed xenograft development by direct antitumor activity through the inhibition of c-MET signaling, at least in part.

## Conclusions

We established a novel CCS cell line called Hewga-CCS and developed a xenograft mouse model. We then demonstrated the direct antitumor effects of pazopanib on Hewga-CCS through the inhibition of HGF/c-MET signaling. Because of the rarity of this disease, Hewga-CCS could be a useful tool for interrogating the tumor biology of CCS and developing new therapeutic strategies.

## Abbreviations

CCS: Clear cell sarcoma; STS: Soft tissue sarcoma; EWS: Ewing sarcoma gene; ATF1: Activating transcription factor 1; TKI: Tyrosine kinase inhibitor; M-MITF: Melanocyte specific-microphthalmia-associated transcription factor; S-100: S100 calcium binding protein; HMB45: Melanoma-associated antigen human melanoma black 45; VEGF: Vascular endothelial growth factor; PDGF: Platelet-derived growth factor; FGF: Fibroblast growth factor; c-Kit: Stem cell factor receptor; M-FISH: Multicolor fluorescence *in situ* hybridization analysis; RTK: Receptor tyrosine kinase; TUNEL: Terminal deoxyribonucleotidyl transferase (TDT)-mediated dUTP-digoxigenin nick end labeling; siRNAs: Small interfering RNAs; HGF: Hepatocyte growth factor.

## Competing interests

The authors had no potential competing interest related to this study.

## Author’s contributions

HO and NN conceived and designed the study, collected, analyzed, and interpreted the data, wrote the manuscript, and provided final approval. TT, TW, YI, KH, and NA collected data. KI collected, analyzed, and interpreted the data. HY provided the study material. All authors read and approved the final manuscript.

## Pre-publication history

The pre-publication history for this paper can be accessed here:

http://www.biomedcentral.com/1471-2407/14/455/prepub

## Supplementary Material

Additional file 1: Figure S1 Clinical course of the patient. A 34-year-old woman with a 3-year history of a slowly growing mass at the 3^rd^ toe of the right foot was referred to our hospital **(A)**. Axial MRI revealed a poorly circumscribed soft tissue mass in the toe, with slightly increased intensity on T1- and T2-weighted images compared with the intensity of muscles **(B)**. While laboratory findings showed no inflammatory reactions, including normal levels of leukocytes (4,310/mm^3^) and CRP (0.1 mg/dl), the initial diagnosis was local paronychia because of unclear border of the mass and the presence of erythema around the nail. Because of persistence of the mass despite oral antibiotic medication, an excisional biopsy was performed. Histopathology showed that the tumors comprised clear cells with large nuclei and distinct nucleoli delineated by fibrous septa into well-defined nests and the patient was diagnosed with clear cell sarcoma. A staging FDG-PET scan of the whole body showed a primary mass in the toe as well as several nodules in the right thigh with increased accumulation of FDG, suggesting metastatic spread to regional lymph nodes (**C**: at presentation, **D**: 5 months later, **E**: 8 months later, **F**: 14 months later). Despite receiving local radiotherapy and three cycles of systemic chemotherapy composed of doxorubicin and ifosfamide, widespread metastatic dissemination to the lymph nodes, bones, skin, spleen, and liver gradually appeared. Twenty-one months after the first presentation, she died because of multiple organ failure. Through the clinical course, the patient has not been exposed to pazopanib. Click here for file

Additional file 2: Figure S2 Growth curve of Hewga-CCS cells. Hewga-CCS cells were cultured in DMEM with 10% FBS. A total of 1 × 10^5^ cells/well were seeded in 6-well plates in triplicate. Cell counts were determined using trypan blue exclusion-based methods. Hewga-CCS cells exhibited logarithmic growth for 8 days, with a doubling time of approximately 44 h in the DMEM with 10% FBS. Click here for file

Additional file 3: Figure S3 A representative G-banded karyotype of the Hewga-CCS cells. The karyotype of the Hewga-CCS cells was 44~47, XX, add(1)(p?36.1),+3,-5,-6,+7,-9, add(11)(q13),-12,-16,+19, add(19)(q?13.1),-20,-22,+mar1,+mar2,+mar3. The arrows indicate chromosomal abnormalities. Click here for file

Additional file 4: Table S1 Chromosome number and cell number of G-band karyotyping. Click here for file

Additional file 5: Table S2 Chromosome number and cell number of M-FISH analysis. Click here for file

Additional file 6: Figure S4 Xenografted mouse model. Hewga-CCS cells (1 × 10^7^) were injected subcutaneously into the flanks of 5-week-old athymic nude mice (BALB/c nu/nu; SLC, Shizuoka, Japan) **(A)**. **(B)** Tumor growth *in vivo*. Tumor size was measured with a caliper, and tumor volume was calculated by the formula (a × b^2^)/2. Click here for file

Additional file 7: Figure S5 Immunohistochemical analyses of Hewga-CCS xenografts. Xenografted mice were treated with 100 mg/kg of pazopanib or vehicle control orally once a day for 1 week, sacrificed 3 h after final administration, and subjected to immunohistochemical analyses. Click here for file

Additional file 8: Figure S6 Secretion of HGF and VEGF from Hewga-CCS cells *in vitro*. Secretion of HGF and VEGF was quantified by ELISA. Hewga-CCS and the synovial sarcoma cell line SYO-1 was cultured in DMEM with 10% FBS for 72 h. The supernatants were subjected to ELISA. Click here for file
