# Supplementary material for: Establishment of a novel clear cell sarcoma cell line (Hewga-CCS), and investigation of the antitumor effects of pazopanib on Hewga-CCS
Source: BMC Cancer. 2014 Jun 19;14:455. doi: 10.1186/1471-2407-14-455 (PMC4076438; doi:10.1186/1471-2407-14-455)
Supplement: Additional file 1: Figure S1 — Clinical course of the patient. A 34-year-old woman with a 3-year history of a slowly growing mass at the 3rd toe of the right foot was referred to our hospital (A). Axial MRI revealed a poorly circumscribed soft tissue mass in the toe, with slightly increased intensity on T1- and T2-weighted images compared with the intensity of muscles (B). While laboratory findings showed no inflammatory reactions, including normal levels of leukocytes (4,310/mm3) and CRP (0.1 mg/dl), the initial diagnosis was local paronychia because of unclear border of the mass and the presence of erythema around the nail. Because of persistence of the mass despite oral antibiotic medication, an excisional biopsy was performed. Histopathology showed that the tumors comprised clear cells with large nuclei and distinct nucleoli delineated by fibrous septa into well-defined nests and the patient was diagnosed with clear cell sarcoma. A staging FDG-PET scan of the whole body showed a primary mass in the toe as well as several nodules in the right thigh with increased accumulation of FDG, suggesting metastatic spread to regional lymph nodes (C: at presentation, D: 5 months later, E: 8 months later, F: 14 months later). Despite receiving local radiotherapy and three cycles of systemic chemotherapy composed of doxorubicin and ifosfamide, widespread metastatic dissemination to the lymph nodes, bones, skin, spleen, and liver gradually appeared. Twenty-one months after the first presentation, she died because of multiple organ failure. Through the clinical course, the patient has not been exposed to pazopanib. [file 1471-2407-14-455-S1.docx]

**Additional file 1**

**Establishment of a novel clear cell sarcoma cell line (Hewga-CCS), and investigation of the antitumor effects of pazopanib on Hewga-CCS**


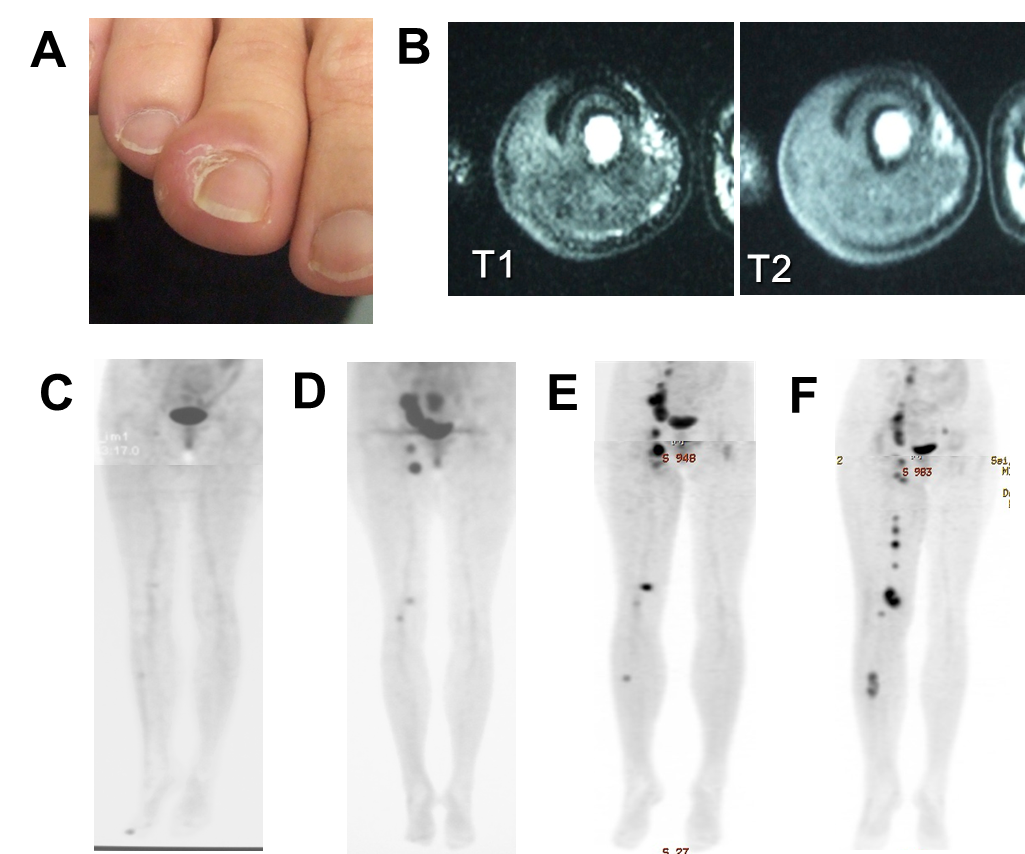


**Figure S1**. Clinical course of the patient

A 34-year-old woman with a 3-year history of a slowly growing mass at the 3^rd^ toe of the right foot was referred to our hospital (A). Axial MRI revealed a poorly circumscribed soft tissue mass in the toe, with slightly increased intensity on T1- and T2-weighted images compared with the intensity of muscles (B). While laboratory findings showed no inflammatory reactions, including normal levels of leukocytes (4,310 /mm^3^) and CRP (0.1 mg/dl), the initial diagnosis was local paronychia because of unclear border of the mass and the presence of erythema around the nail. Because of persistence of the mass despite oral antibiotic medication, an excisional biopsy was performed. Histopathology showed that the tumors comprised clear cells with large nuclei and distinct nucleoli delineated by fibrous septa into well-defined nests and the patient was diagnosed with clear cell sarcoma. A staging FDG-PET scan of the whole body showed a primary mass in the toe as well as several nodules in the right thigh with increased accumulation of FDG, suggesting metastatic spread to regional lymph nodes (C: at presentation, D: 5 months later, E: 8 months later, F: 14 months later). Despite receiving local radiotherapy and three cycles of systemic chemotherapy composed of doxorubicin and ifosfamide, widespread metastatic dissemination to the lymph nodes, bones, skin, spleen, and liver gradually appeared. Twenty-one months after the first presentation, she died because of multiple organ failure. Through the clinical course, the patient has not been exposed to pazopanib.
