# Supplementary material for: Establishment of a novel clear cell sarcoma cell line (Hewga-CCS), and investigation of the antitumor effects of pazopanib on Hewga-CCS
Source: BMC Cancer. 2014 Jun 19;14:455. doi: 10.1186/1471-2407-14-455 (PMC4076438; doi:10.1186/1471-2407-14-455)
Supplement: Additional file 2: Figure S2 — Growth curve of Hewga-CCS cells. Hewga-CCS cells were cultured in DMEM with 10% FBS. A total of 1 × 10^5^ cells/well were seeded in 6-well plates in triplicate. Cell counts were determined using trypan blue exclusion-based methods. Hewga-CCS cells exhibited logarithmic growth for 8 days, with a doubling time of approximately 44 h in the DMEM with 10% FBS. [file 1471-2407-14-455-S2.doc]

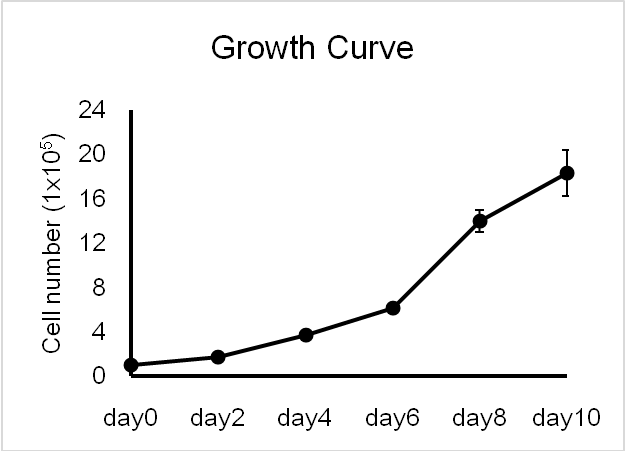


**Figure S2**. Growth curve of Hewga-CCS cells

Hewga-CCS cells were cultured in DMEM with 10% FBS. A total of 1 × 10^5^ cells/well were seeded in 6-well plates in triplicate. Cell counts were determined using trypan blue exclusion-based methods. Hewga-CCS cells exhibited logarithmic growth for 8 days, with a doubling time of approximately 44 h in the DMEM with 10% FBS.
