# Supplementary material for: Establishment of a novel clear cell sarcoma cell line (Hewga-CCS), and investigation of the antitumor effects of pazopanib on Hewga-CCS
Source: BMC Cancer. 2014 Jun 19;14:455. doi: 10.1186/1471-2407-14-455 (PMC4076438; doi:10.1186/1471-2407-14-455)
Supplement: Additional file 3: Figure S3 — A representative G-banded karyotype of the Hewga-CCS cells. The karyotype of the Hewga-CCS cells was 44~47, XX, add(1)(p?36.1),+3,-5,-6,+7,-9, add(11)(q13),-12,-16,+19, add(19)(q?13.1),-20,-22,+mar1,+mar2,+mar3. The arrows indicate chromosomal abnormalities. [file 1471-2407-14-455-S3.doc]

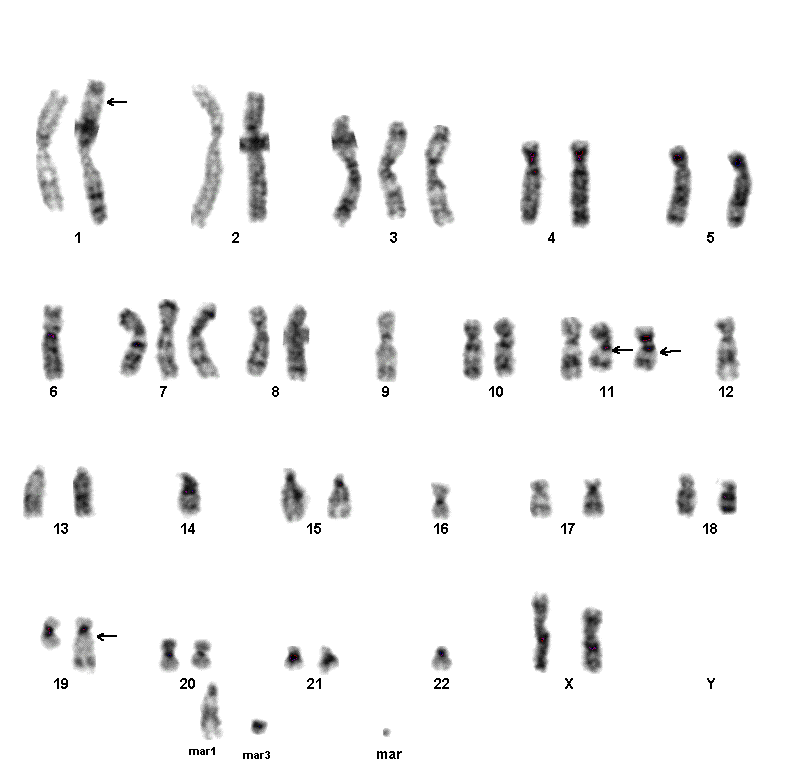


**Figure S3**. A representative G-banded karyotype of the Hewga-CCS cells

The karyotype of the Hewga-CCS cells was 44~47, XX, add(1)(p?36.1),+3,-5,-6,+7,-9, add(11)(q13),-12,-16,+19, add(19)(q?13.1),-20,-22,+mar1,+mar2,+mar3. The arrows indicate chromosomal abnormalities.
