# Supplementary material for: Establishment of a novel clear cell sarcoma cell line (Hewga-CCS), and investigation of the antitumor effects of pazopanib on Hewga-CCS
Source: BMC Cancer. 2014 Jun 19;14:455. doi: 10.1186/1471-2407-14-455 (PMC4076438; doi:10.1186/1471-2407-14-455)
Supplement: Additional file 4: Table S1 — Chromosome number and cell number of G-band karyotyping. [file 1471-2407-14-455-S4.doc]

**Table S1**. Chromosome number and cell number of G-band karyotyping

| Chromosome number | 44 | 45 | 46 | 47 |
| --- | --- | --- | --- | --- |
| Cell number | 3 | 19 | 25 | 3 |
