# Supplementary material for: Establishment of a novel clear cell sarcoma cell line (Hewga-CCS), and investigation of the antitumor effects of pazopanib on Hewga-CCS
Source: BMC Cancer. 2014 Jun 19;14:455. doi: 10.1186/1471-2407-14-455 (PMC4076438; doi:10.1186/1471-2407-14-455)
Supplement: Additional file 6: Figure S4 — Xenografted mouse model. Hewga-CCS cells (1 × 10^7^) were injected subcutaneously into the flanks of 5-week-old athymic nude mice (BALB/c nu/nu; SLC, Shizuoka, Japan) (A). (B) Tumor growth in vivo. Tumor size was measured with a caliper, and tumor volume was calculated by the formula (a × b2)/2. [file 1471-2407-14-455-S6.doc]

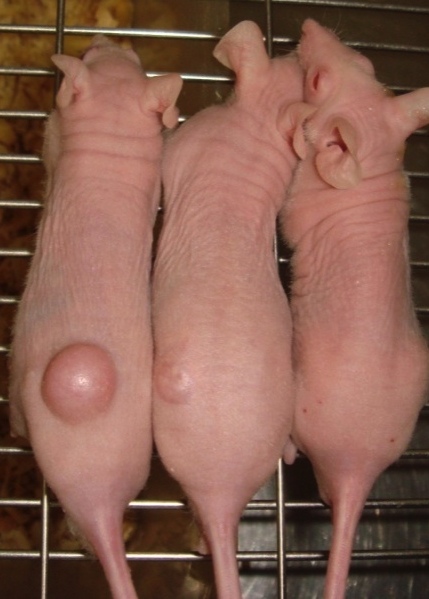

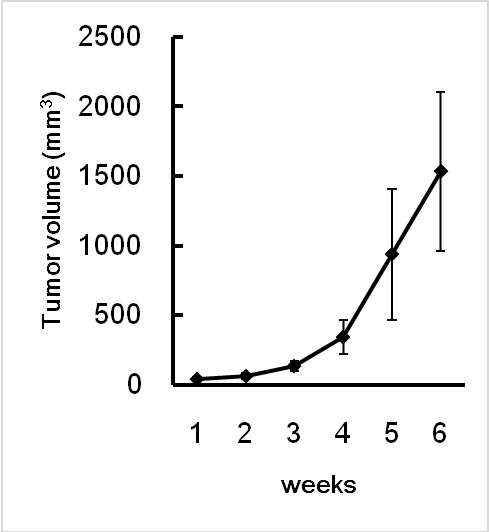


**B**

**Figure S4**. Xenografted mouse model

Hewga-CCS cells (1 × 10^7^) were injected subcutaneously into the flanks of 5-week-old athymic nude mice (BALB/c nu/nu; SLC, Shizuoka, Japan) (A).

(B) Tumor growth *in vivo*

Tumor size was measured with a caliper, and tumor volume was calculated by the formula (a × b^2^)/2.
