# Supplementary material for: Establishment of a novel clear cell sarcoma cell line (Hewga-CCS), and investigation of the antitumor effects of pazopanib on Hewga-CCS
Source: BMC Cancer. 2014 Jun 19;14:455. doi: 10.1186/1471-2407-14-455 (PMC4076438; doi:10.1186/1471-2407-14-455)
Supplement: Additional file 7: Figure S5 — Immunohistochemical analyses of Hewga-CCS xenografts. Xenografted mice were treated with 100 mg/kg of pazopanib or vehicle control orally once a day for 1 week, sacrificed 3 h after final administration, and subjected to immunohistochemical analyses. [file 1471-2407-14-455-S7.doc]

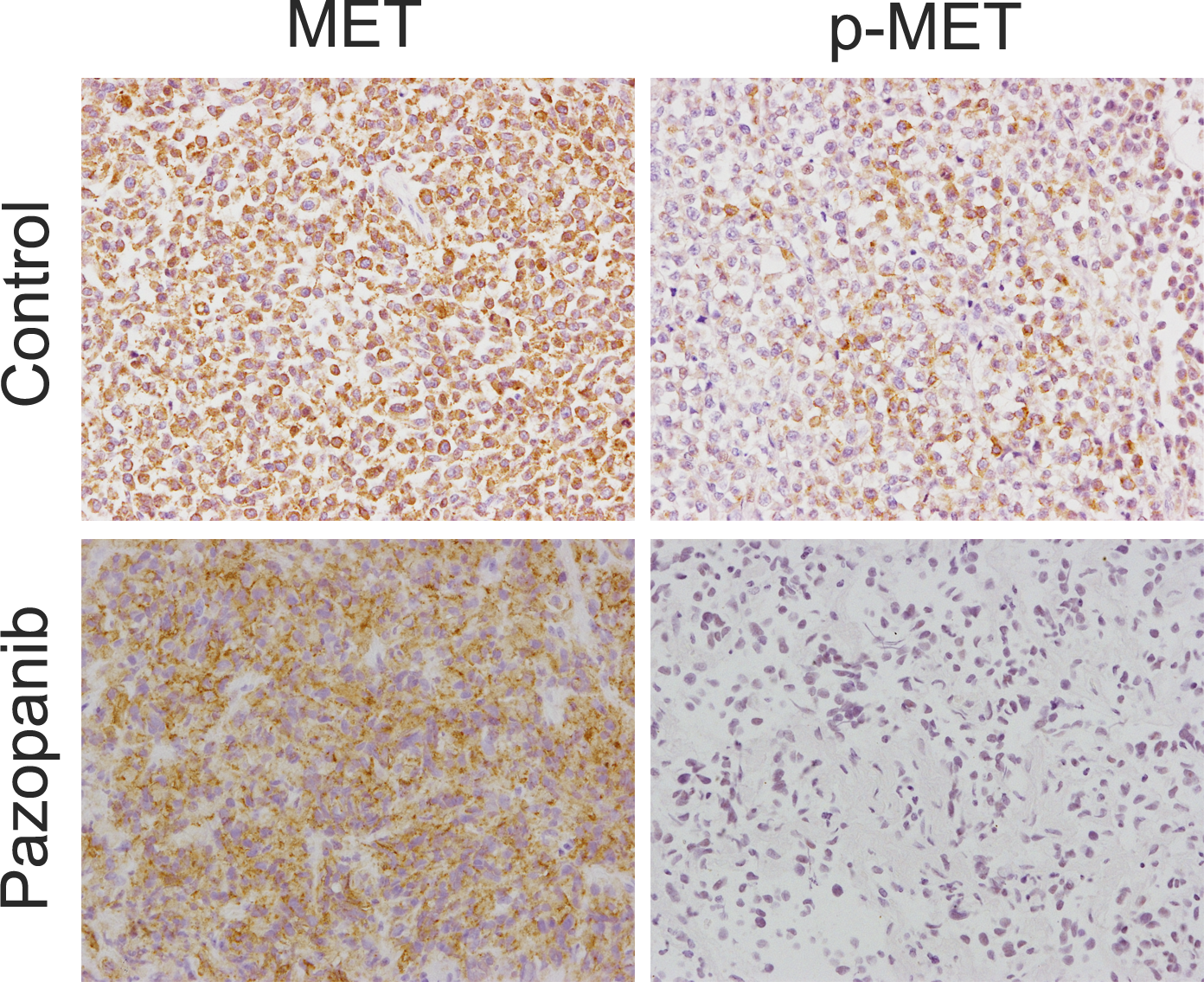


**Figure S5**. Immunohistochemical analyses of Hewga-CCS xenografts

Xenografted mice were treated with 100 mg/kg of pazopanib or vehicle control orally once a day for 1 week, sacrificed 3 h after final administration, and subjected to immunohistochemical analyses.
