# Supplementary material for: Establishment of a novel clear cell sarcoma cell line (Hewga-CCS), and investigation of the antitumor effects of pazopanib on Hewga-CCS
Source: BMC Cancer. 2014 Jun 19;14:455. doi: 10.1186/1471-2407-14-455 (PMC4076438; doi:10.1186/1471-2407-14-455)
Supplement: Additional file 8: Figure S6 — Secretion of HGF and VEGF from Hewga-CCS cells in vitro. Secretion of HGF and VEGF was quantified by ELISA. Hewga-CCS and the synovial sarcoma cell line SYO-1 was cultured in DMEM with 10% FBS for 72 h. The supernatants were subjected to ELISA. [file 1471-2407-14-455-S8.doc]

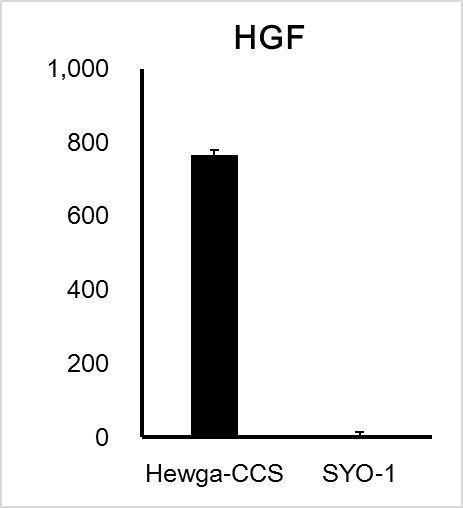

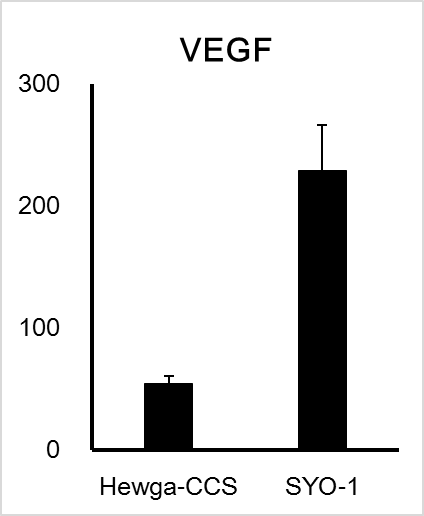


(pg/ml)

(pg/ml)

**Figure S6**. Secretion of HGF and VEGF from Hewga-CCS cells *in vitro*

Secretion of HGF and VEGF was quantified by ELISA. Hewga-CCS and the synovial sarcoma cell line SYO-1 was cultured in DMEM with 10% FBS for 72 h. The supernatants were subjected to ELISA.
